# A quantitative proteomic analysis of the molecular mechanism underlying fertility conversion in thermo-sensitive genetic male sterility line AnnongS-1

**DOI:** 10.1186/s12870-019-1666-5

**Published:** 2019-02-11

**Authors:** Siyao Wang, Qingyuan Tian, Shiqi Zhou, Dandan Mao, Liangbi Chen

**Affiliations:** College of Life Science, Hunan Normal University, Changsha, 410081 Hunan China

**Keywords:** Thermo-sensitive genetic male-sterility, AnnongS-1, Proteomics, Fertility conversion, Hybrid rice

## Abstract

**Background:**

Thermo-sensitive genetic male sterile (TGMS) lines have been widely used in two-line hybrid rice breeding. The two-line hybrids have increased rice yields substantially. However, the effect of environmental temperatures on the fertility conversion is still not fully clear. In this study, we performed a tandem mass tag (TMT)-based proteomic analysis on the anthers of the TGMS line AnnongS-1 grown under permissive (low) temperature (21 °C) and restrictive (high) temperature (> 26 °C) conditions in an attempt to explore the effect of temperature on the fertility of the male sterile line.

**Results:**

After the AnnongS-1 plants were induced under either permissive or restrictive conditions, morphological observations and I_2_-KI staining confirmed that the pollen grains formed under high temperature conditions were abortive while those formed under low temperature developed normally. In comparison to the plants grown under permissive conditions, the restrictive high-temperature conditions led to the differential accumulation of 89 proteins in the anthers, of which 46 were increased in abundance and 43 were decreased in abundance. Most of the subcellular compartments of the anther cells had one or more proteins that had been differentially accumulated, with the cytoplasm and chloroplast having the greatest accumulations. More than 40% of the differentially abundant proteins (DAPs) were enzymes involved in photosynthesis, energy metabolism, biosynthesis and catabolism of cellular components, metabolic regulation, defense and stress, etc. The DAPs related to protein metabolism accounted for the largest proportion (21.35%), followed by those related to defense and stress (12.36%), metabolic regulation (10.11%) and carbohydrate metabolism (8.99%), indicating that such biological processes in anther cells were more susceptible to high temperature stress.

**Conclusions:**

The restrictive temperature induction caused fertility-sterility conversion in the TGMS line AnnongS-1 mainly by adversely affecting the metabolism of protein, carbohydrate and energy, and decreasing the abundances of important proteins closely related to defense and stress, thereby impeding the growth and development of the pollen and weakening the overall defense and ability to endure stress of AnnongS-1. These data are helpful for deepening our understanding of the molecular mechanism underlying fertility conversion in TGMS lines.

**Electronic supplementary material:**

The online version of this article (10.1186/s12870-019-1666-5) contains supplementary material, which is available to authorized users.

## Background

Rice (*Oryza sativa* L*.*) is the staple food for more than half of the world’s population and is extensively cultivated, particularly in Asia [1, 2]. As the world population continues to grow, improving rice productivity to meet increasing food demands is a huge challenge. A proven strategy to meet this demand is the development of hybrid rice. Hybrid rice is mainly based on three-line and two-line male-sterility systems [3, 4]. The three-line system consists of a male sterile line, a maintainer line and a restorer. Although the three-line hybrid rice is a major type of hybrid rice [3, 5], the limited germplasm of restorer lines and the genetic diversity between cytoplasmic male-sterility lines and restorer lines have limited further improvements in three-line hybrid rice breeding [6, 7]. A number of genetic male sterility mutations are regulated by environmental factors such as photoperiod and/or temperature; the plants are male sterile under restrictive conditions (high temperature and/or long day) and fertile under permissive conditions (low temperature and/or short day). That is to say, the environmentally sensitive genetic male-sterility line serves as both the male sterile line and the maintainer line under different environmental conditions [2]. This has provided opportunities for the development of two-line breeding systems.

To date, several temperature-sensitive male sterile mutants have been used in rice breeding programs, including the AnnongS-1 [7, 8], Zhu 1S [9], HengnongS-1 [8, 10], Xian S [11], and others. AnnongS-1-derived thermo-sensitive genetic male sterile (TGMS) lines have been widely used in two-line hybrid rice breeding [7, 12]. A recent series of genetic and biochemical studies have provided insight into the relevant molecular mechanisms [2, 12]. According to previous studies, fertility alteration occurs during the phase from formation of the pollen mother cell to the tetrad formed by meiosis [13]. Moreover, Zhou et al. [7] found that mutation of the *thermosensitive genetic male sterile 5* (*tms5*) gene in AnnongS-1 causes the TGMS trait through a loss of RNase Z (s1) function. RNase Z (s1) cleaves *ubiquitin fusion ribosomal protein L40* (*Ub*_*L40*_) mRNAs. In *tms5* mutants no RNase Z (s1) is produced and high temperature results in overaccumulation of *Ub*_*L40*_ mRNAs, which leads to defective pollen production and male sterility. Furthermore, Zhou et al. demonstrated that transgenic plants that overexpressed *Ub*_*L40*_*1*- and *Ub*_*L40*_*4* produced higher levels of *Ub*_*L40*_*1* and *Ub*_*L40*_*4* mRNAs and exhibited partial pollen abortion. *Ub*_*L40*_*1* and *Ub*_*L40*_*4* knockdown plants exhibited reduced levels of *Ub*_*L40*_*1* and *Ub*_*L40*_*4* mRNAs and partially restored male fertility. However, they did not demonstrate whether the level of Ub_L40_ proteins increased when *Ub*_*L40*_ mRNAs were overaccumulated, lacking experimental evidence on fertility conversion mechanism at the protein level. We speculate that male sterility is not completely determined by *Ub*_*L40*_ mRNAs because RNase Z(s1) may also cleave other mRNAs so that other related proteins and cellular processes are affected.

Contemporary proteomics provides a powerful high-throughput means for identifying key proteins involved with male-sterility pathways and for identifying the relevant molecular mechanisms. Xiao et al. [14] have used a strategy involving an SDS-PAGE (sodium dodecyl sulfate-polyacrylamide gel electrophoresis) combined with MALDI-TOF (matrix-assisted laser desorption/ionization-time of flight) mass spectrometry to comparatively analyze the young panicle proteome in TGMS rice Zhu 1S under sterile and fertile conditions. They identified 20 differentially abundant proteins (DAPs) and found that the proteins are mainly involved with energy metabolism, protein synthesis, cell wall formation, stress response, and other cellular processes during pollen development, thereby suggesting the critical roles that the proteins play during fertility conversion in rice. More recently, the young panicle proteomes of two TGMS rice lines Zhu 1S and Zhun S were comparatively analyzed [15]. The identified proteins are involved with 16 metabolic pathways and cellular processes; compared with Zhun S, Zhu 1S has lower levels of ROS scavengers, indole-3-acetic acid and soluble proteins in the young panicles. These data have improved our understanding of the mechanism underlying fertility alterations in TGMS lines, but not how environmental temperature regulates fertility-sterility conversion, which remains unclear. In the present study, we performed a quantitative proteomic analysis on the anthers of AnnongS-1 in an attempt to probe into the molecular mechanism underlying fertility-sterility conversion under different temperatures. AnnongS-1 plants were induced under low (21 °C) or high (> 26 °C) temperatures. After comparative morphological observations and the I_2_-KI staining of pollen grains, a TMT labeling-based method was employed to quantitatively analyze the anther proteomes of AnnongS-1 treated under different temperatures. Eighty-nine DAPs were identified and the RNAs of several representative DAPs were further analyzed by quantitative RT-PCR (reverse transcription-polymerase chain reaction). We describe the molecular mechanisms underlying the temperature-induced fertility conversion based on our experimental results and relevant reports from the literature.

## Results

### Morphological characteristics

**Fig. 1:**
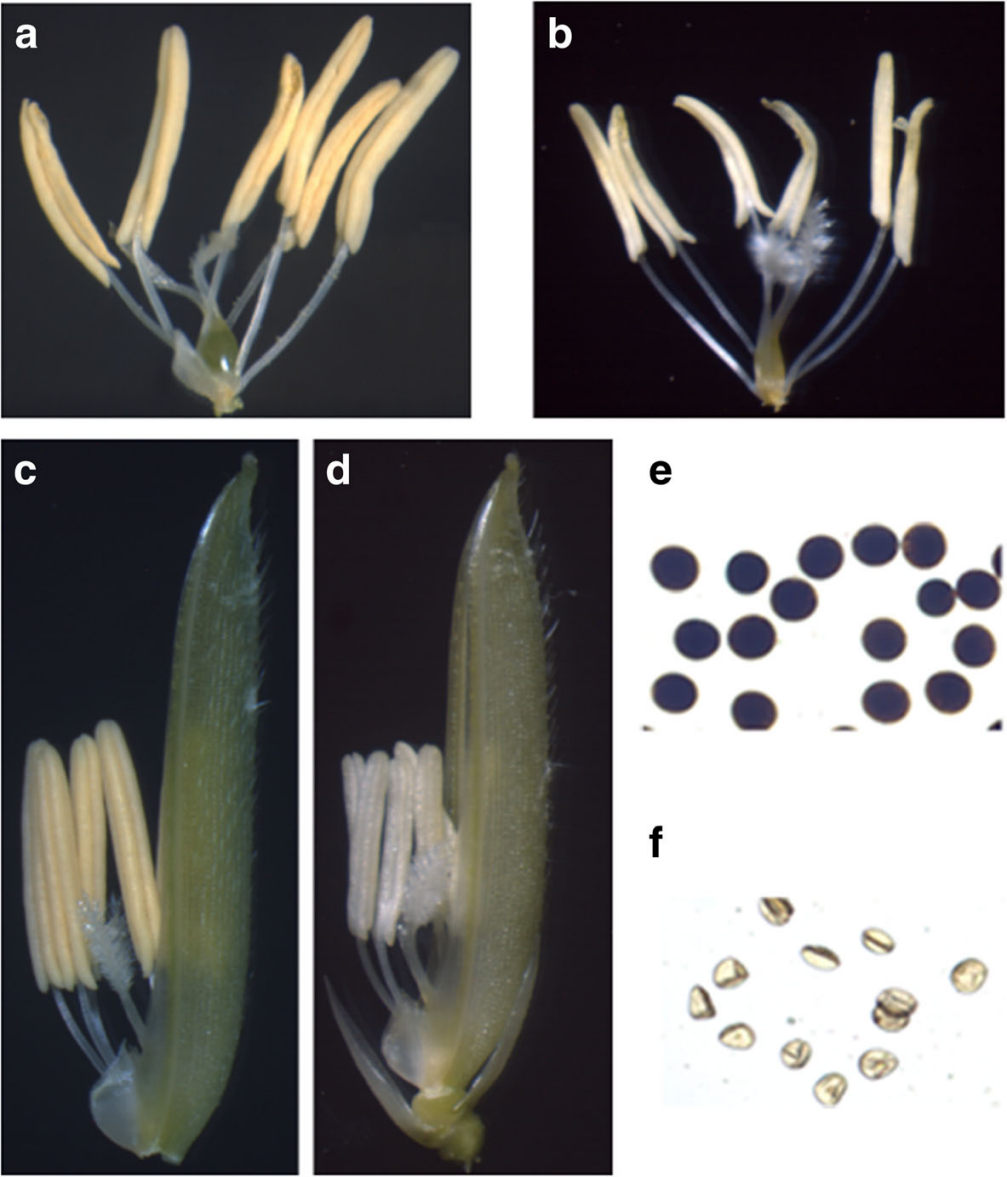
Comparative phenotypes of florets of the AnnongS-1 formed under low and high temperatures. **a**: low temperature-formed stamens; **b**: high temperature-formed stamens; **c**: low temperature-formed stamens and inner palea; **d**: high temperature-formed stamens and inner palea; **e**: I_2_-KI-stained pollen grains of the anthers formed under low temperature; **f**: I_2_-KI-stained pollen grains of the anthers formed under high temperature

In the plants grown for 6 days at 21 °C, the matured stamens of the florets were strong and golden yellow in color, with a green inner palea (Fig. 1a and c); the anthers contained a large number of spherical pollen grains when viewed under light microscopy and were darkly stained by I_2_-KI, indicating that the pollen grains were rich in starch (Fig. 1e). On the contrary, in the plants grown for 6 days at > 26 °C, the stamens of the florets were clearly slenderer than the low temperature-grown stamens and had light yellow to yellowish white colors, with a light green inner palea (Fig. 1b and d). The anthers contained fewer pollen grains, which were not only wizened and smaller than the fertile pollen grains, but also were unstainable by I_2_-KI, suggesting that the pollen grains contained almost no starch (Fig. 1f). These observations of morphology and I_2_-KI staining confirmed that the pollen grains formed under high temperature conditions were abortive while those formed under low temperature developed normally, and the anthers would be suitable for analysis of DAPs.

### Quantitative analysis of anther proteomes

**Fig. 2:**
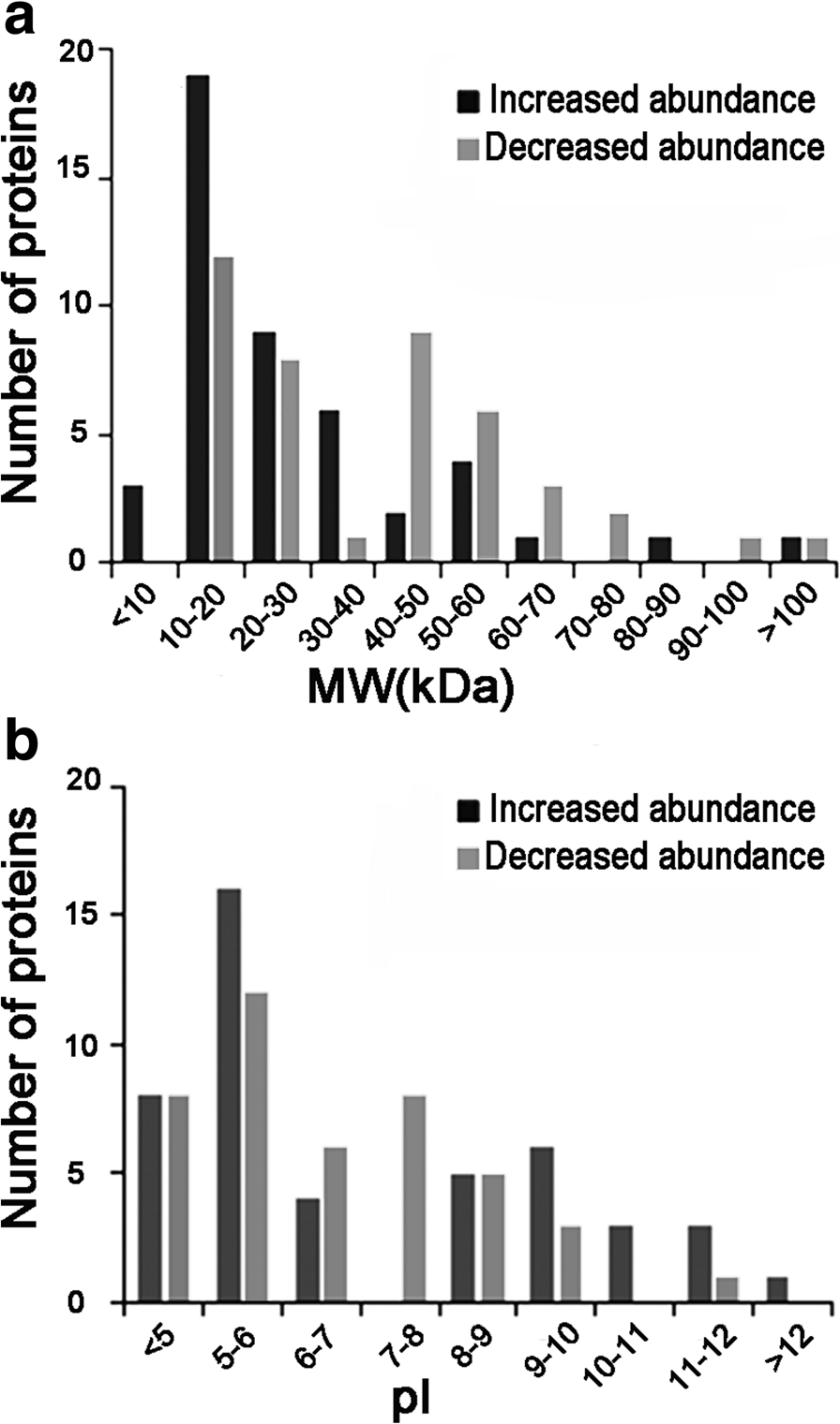
Distributions of DAPs as a function of molecular weight (**a**) and isoelectric point (**b**)

The quantitative analysis was performed in triplicate which identified 1040, 1058 and 736 proteins, respectively. The proteins with an average fold-change in abundance greater than ±1.5 (*p < 0.05*) between the test and the control were considered DAPs. Based on this criterion, 89 DAPs were identified; 46 were increased in abundance and 43 decreased in abundance in the high temperature-formed sterile anthers, indicating that the fertility-sterility conversion in the TGMS line AnnongS-1 involved an increase or decrease in the abundances of approximately equal numbers of proteins under our experimental conditions. The comparative analysis indicated that the molecular weight (MW) of the proteins with increased abundance ranged widely, from 5.5 kDa to 116.6 kDa, slightly greater than that of the proteins with decreased abundance (11.1–106.2 kDa). The distribution profiles of the two groups of proteins were similar, with the 20–30 kDa proteins accounting for the largest proportion (Fig. 2a and Additional file 1: Table S1). There were no obvious differences in the distributions of the isoelectric points (pI) between the proteins with increased or decreased abundance (Fig. 2b and Additional file 1: Table S1).These data demonstrate that there are no significant differences in the molecular size and acid-base properties between the two groups of high temperature-induced DAPs.

### Gene ontology analysis of the differentially abundant proteins

**Fig. 3:**
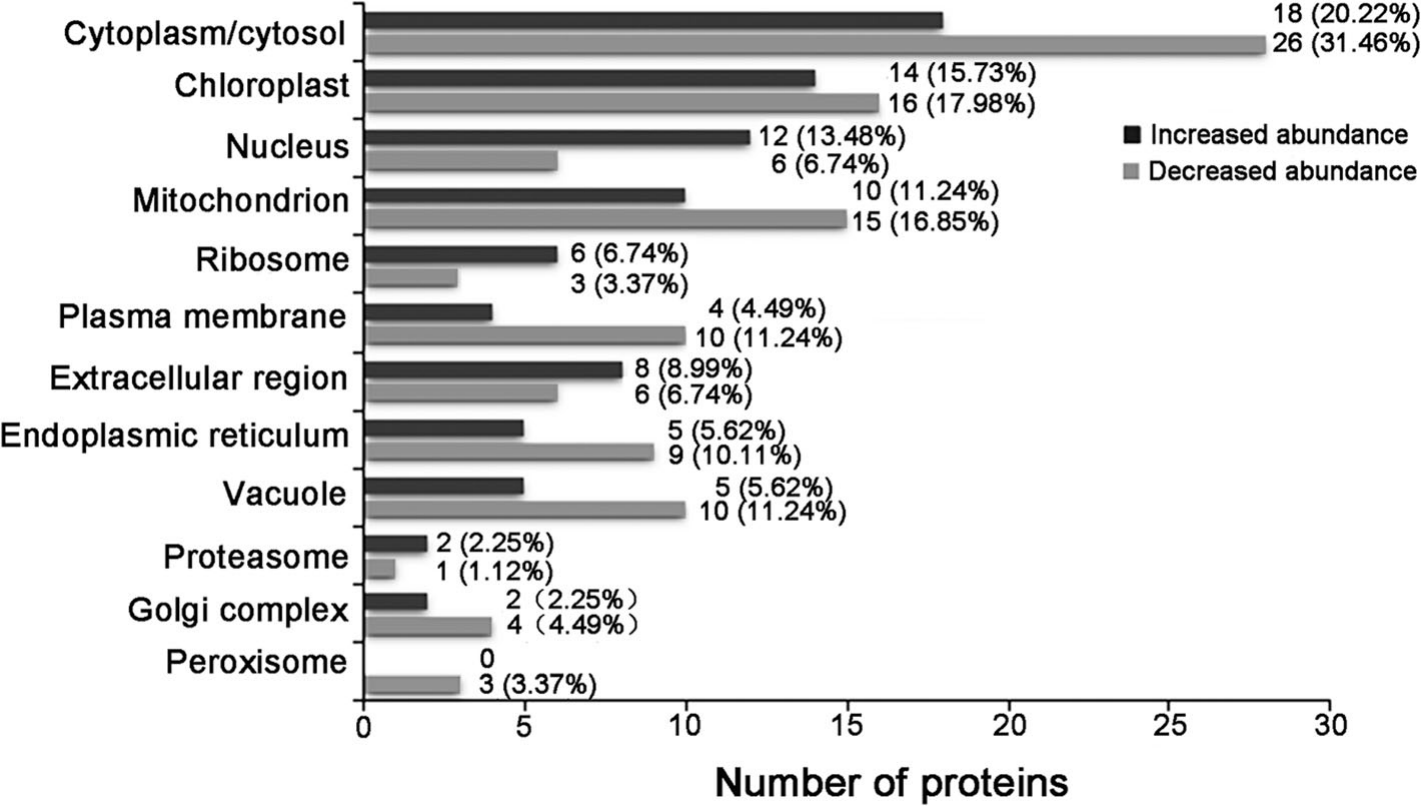
Subcellular distribution of the DAPs

According to the universal GO cellular component annotations (Additional file 1: Table S1), we analyzed the subcellular distribution of the identified DAPs for twelve subcellular compartments: cytoplasm, chloroplast, mitochondrion, nucleus, ribosome, plasma membrane, endoplasmic reticulum, Golgi complex, vacuole, proteasome, peroxisome and the extracellular region. As shown in Fig. 3 and Additional file 1: Table S1, each DAP had localization in one or more subcellular compartments. Cytoplasm was the main subcellular localization for the DAPs, with 46 (accounting for 51.68% of 89 DAPs) being distributed in this subcellular compartment, followed by chloroplast (30, 33.71%), mitochondrion (25, 28.09%) and nucleus (18, 20.22%), demonstrating that the proteins in these compartments were more susceptible to high temperature. Comparative analysis showed that there were certain differences between the distribution profiles of the two groups of DAPs. Clear differences in the distributions of the DAPs occurred mainly in the nucleus, ribosome, plasma membrane, endoplasmic reticulum, vacuole, proteasome, Golgi complex, and peroxisome. These differences in distribution suggest that high temperature affects the subcellular compartments with changes in abundance levels of proteins, and thus the functions of those compartments to some extent.

**Fig. 4:**
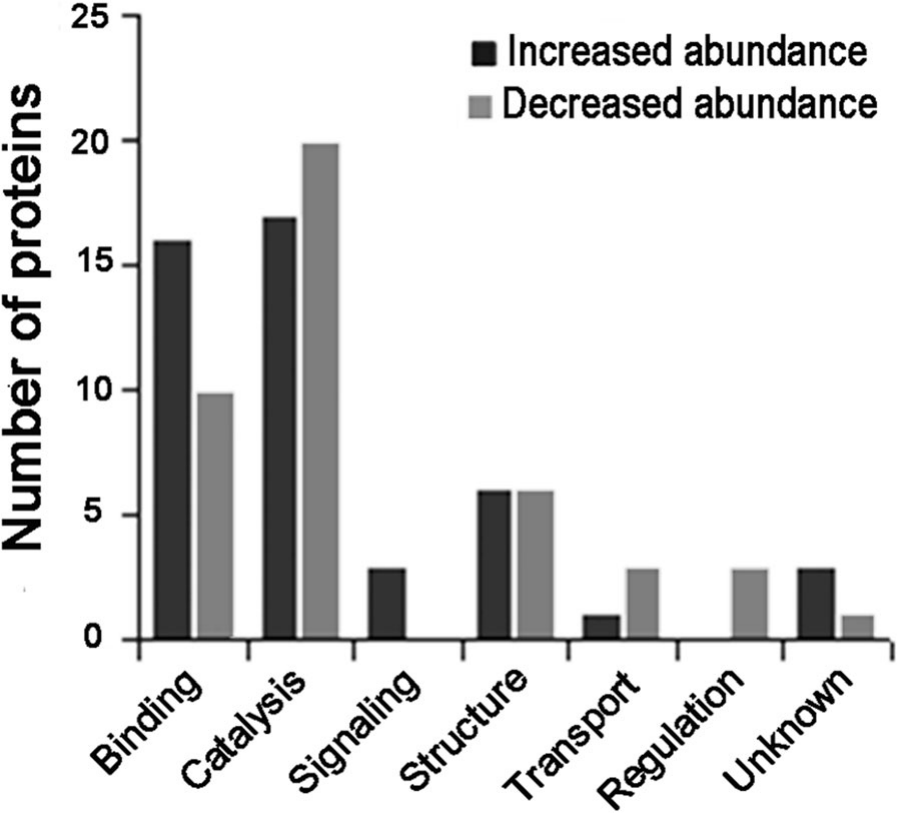
Classification of molecular functions of the DAPs

To understand the functions of the DAPs, we analyzed the proteins with increased or decreased abundance with respect to molecular functions. Since a protein usually has multiple biological functions, for the convenience of comparative analysis, we categorized the DAPs into seven categories according to their representative functions: binding, catalysis, structure, transport, signaling, regulation, and unknown (Fig. 4). As seen in Fig. 4 and Additional file 1: Table S1, more than 40% (37, 41.57%) of the 89 DAPs were enzymes having catalytic functions, such as ribulose-bisphosphate carboxylase, succinate dehydrogenase, NADH dehydrogenase, ATP synthase, protein disulfide oxidoreductase, peptidyl-prolyl cis-trans isomerase, RNA polymerase, protein kinase, GTPase, glutathione transferase, peroxidase, superoxide dismutase, and others. Some were increased in abundance while others were decreased in abundance. Theses enzymes are mainly involved in photosynthesis, biosynthesis, catabolism, energy metabolism, metabolic regulation, defense, stress, and other processes, suggesting that high temperature increased or decreased the abundance levels of multiple key enzymes and thus altered the relevant cellular processes in anthers. Changes in the abundance levels of certain enzymes were the primary way for high temperature to convert the fertility of the anthers. “Binding” was the second largest category after “Catalyst”; the DAPs with binding functions accounted for 29.21%. These proteins could bind multiple types of substances, such as proteins, lipids, nucleic acids, ATP, steroids and metal ions, thereby affecting the related cellular processes. The proteins in the “Structure” category accounted for 13.4%, which indicated that high temperature also had a great impact on the cellular structure of the anthers. Besides, high temperature decreased the abundance levels of a few proteins in signaling, transport and regulation. In addition, four DAPs lacked GO annotation on molecular function, and BLAST analysis indicated that no proteins showed high homology with them. Therefore, they were categorized as “Unknown”.

**Fig. 5:**
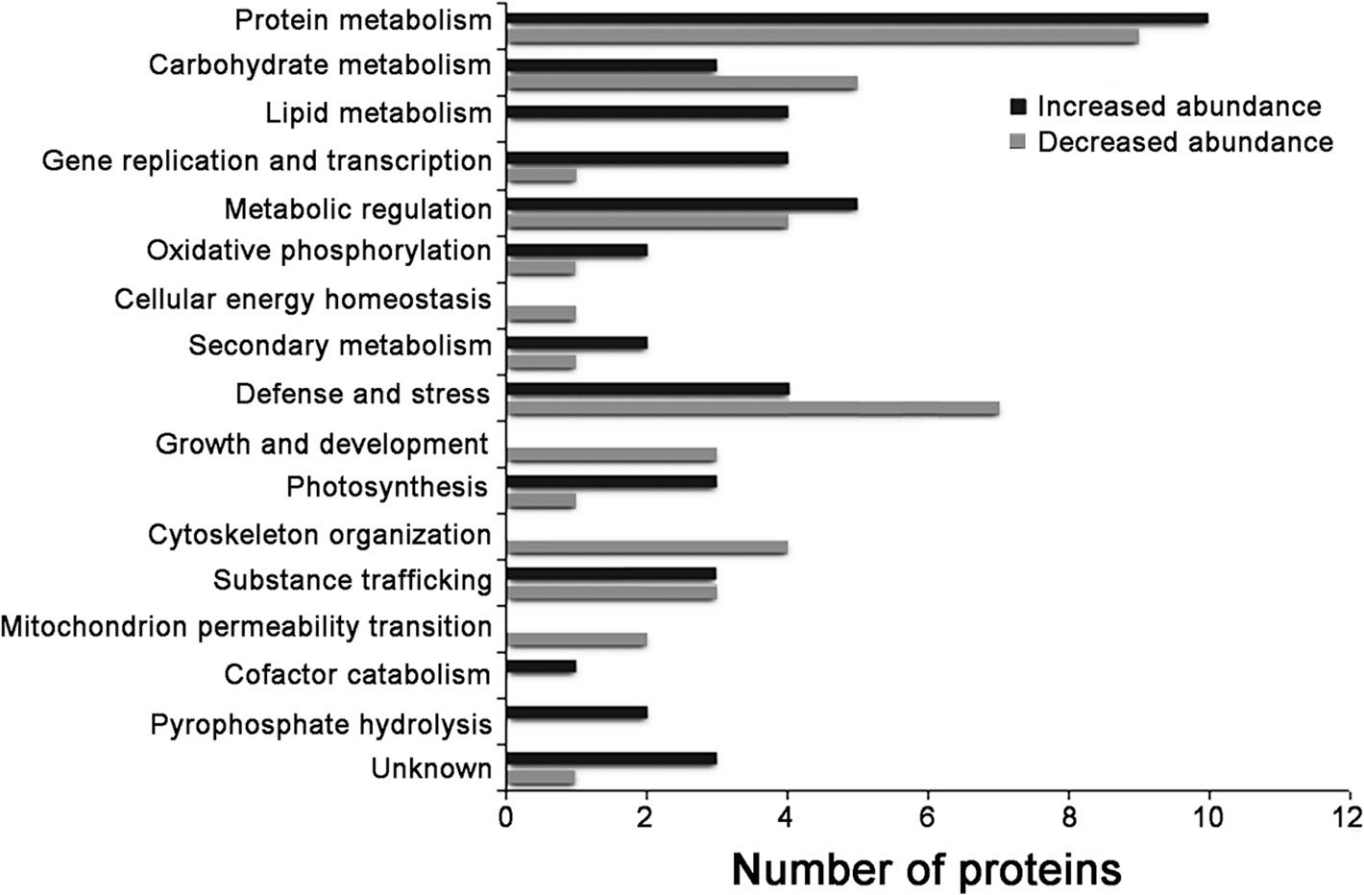
Biological processes involved in the DAPs

The biological processes of the DAPs were analyzed using GO annotations. Nearly 200 distinct biological process GO terms were obtained (Additional file 1: Table S1). We categorized these biological processes into seventeen groups according to the functional similarity of these processes: protein metabolism, carbohydrate metabolism, lipid metabolism, gene replication and transcription, metabolic regulation, oxidative phosphorylation, cellular energy homeostasis, secondary metabolism, defense and stress, growth and development, photosynthesis, cytoskeleton organization, substance trafficking, mitochondrial permeability transition, cofactor catabolism, pyrophosphate hydrolysis and Unknown (Fig. 5). Of the 89 DAPs, the proteins related to protein metabolism accounted for the largest proportion (19, 21.35%), followed by the proteins related to defense and stress (11, 12.36%), metabolic regulation (9, 10.11%) as well as carbohydrate metabolism (8, 8.99%), demonstrating that these biological processes in anther cells were more easily affected by high temperature stress because of the number of affected proteins.

Figure 5 also shows that only proteins with increased abundance were involved with the cellular processes of lipid metabolism, cofactor catabolism and pyrophosphate hydrolysis, suggesting that these processes were enhanced, whereas the cellular processes of cell energy homeostasis, growth and development, cytoskeleton organization, and mitochondrion permeability transition were weakened. Only proteins with decreased abundance were involved with the latter processes. In the remaining cellular processes, there were both increased and decreased abundance proteins, and the overall effect of high temperature would be determined by the relative importance and differential folds of the proteins in the related cellular processes.

The proteins that were most affected by high temperature stress are shown in Table 1. Cellular processes with DAPs in the top ten with increased abundance (but not in the top ten with decreased abundance) were protein phosphorylation in metabolism regulation (Os05g0480000 protein), lipid transport in lipid metabolism (non-specific lipid-transfer protein 4), nucleosome assembly in DNA replication (histone-like protein), and mRNA transcription (Os09g0110400 protein), indicating that these processes were more active under high temperature stress. Conversely, the cellular processes with DAPs in the top ten with decreased abundance were cytoskeleton organization, cellular energy homeostasis and cellular energy homeostasis, indicating that high temperature stress impeded these processes by decreasing the abundance levels of the key proteins or enzymes. Other cellular processes, such as protein and carbohydrate metabolism, defense and stress, had DAPs in both top ten lists. Overall changes caused by high temperature stress would be determined by the relative importance and differential folds of the DAPs (see Discussion).

**Table 1:** The top ten DAPs with increased abundance and the top ten DAPs with decreased abundance

Protein name	HT/LT^*a*^	Biological process	Classification
DAPs with increased abundance			
Os05g0480000 protein	2.924	Protein phosphorylation; photosystem II stabilization	Metabolic regulation
Ribosomal protein L37	2.674	Translation	Protein metabolism
Non-specific lipid-transfer protein 4	2.525	Transport; lipid transport.	Lipid metabolism
Os09g0513000 protein	2.421	Protein targeting to chloroplast	Substance trafficking
Histone-like protein	2.370	Nucleosome assembly	Gene replication and transcription
Glycosyltransferase	2.315	Metabolic process	Secondary metabolism
Cytochrome c oxidase subunit 6b	2.309	Response to salt stress	Oxidative phosphorylation
Os09g0110400 protein	2.288	Transcription from RNA polymerase II promoter	Gene replication and transcription
DNA-damage-repair/toleration protein-like	2.273	Carbohydrate metabolic process; response to cold	Carbohydrate metabolism
Os12g0405700 protein	2.273	Response to cytokinin; defense and stress	Defense and stress
DAPs with decreased abundance			
Glycosyl hydrolase family 3 N terminal	0.282	Carbohydrate metabolic process, glucan catabolic process	Carbohydrate metabolism domain containing protein, expressed
DnaK protein	0.336	Endoplasmic reticulum stress response; pollen tube growth	Defense and stress
Os01g0933900 protein	0.380	Glutathione metabolic process	Defense and stress
Tubulin beta-1 chain	0.396	Cytoskeleton organization; microtubule-based process	Cytoskeleton organization
Carboxypeptidase	0.410	Proteolysis	Protein metabolism
Peroxidase	0.441	Response to oxidative stress; cellular oxidant detoxification	Defense and stress
Adenylate kinase 3	0.458	Nucleobase-containing compound metabolic process	Cellular energy homeostasis
Os07g0194000 protein	0.464	Exocytosis; vesicle fusion; vesicle-mediated transport	Substance trafficking
Os03g0200500 protein	0.472	Translation	Protein metabolism
Translationally-controlled tumor protein homolog	0.479	Cell differentiation	Growth and development

^a^, Relative protein levels under high temperature (HT)/under low temperature (LT). DAPs, differentially abundant proteins

### mRNA levels of representative DAPs

**Fig. 6:**
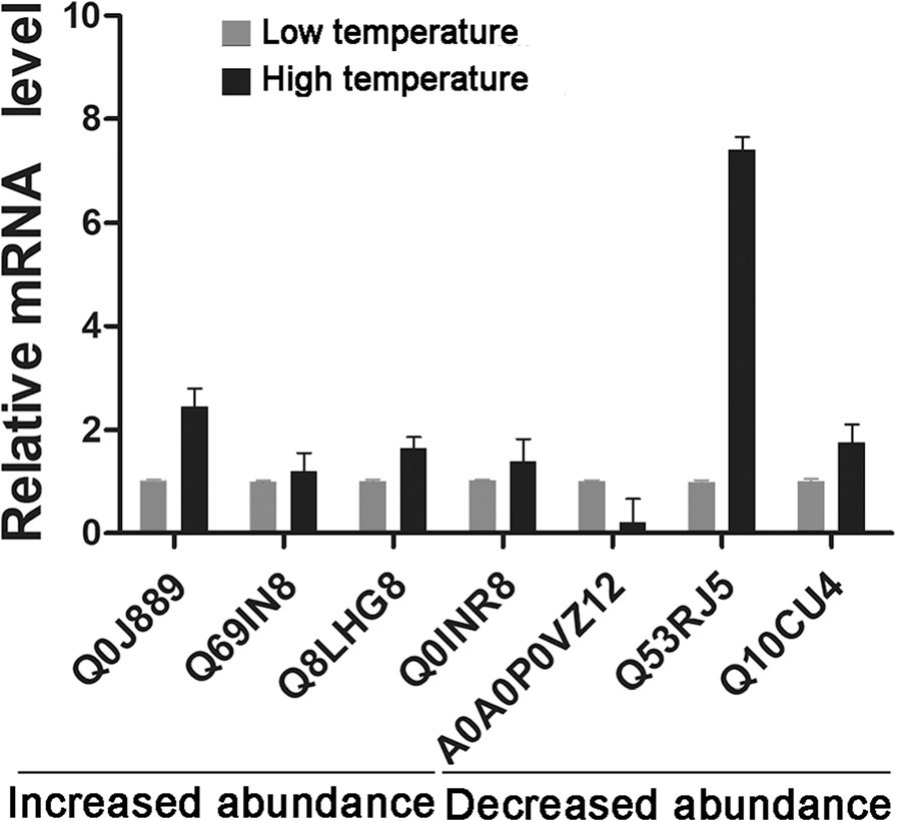
Relative mRNA levels of selected DAPs in the anthers formed under high and low temperatures. The mRNA expression levels in the anthers formed under high temperature were normalized to control (formed under low temperature)

To validate the expression of the DAPs at the transcriptional level, qPCR (quantitative polymerase chain reaction) was employed to analyze the mRNA level of the DAPs selected mainly from the protein sets involved with protein and carbohydrate metabolism and defense and stress because they were the DAPs most affected by high temperature. A total of seven proteins, four with increased abundance and three with decreased abundance were selected. The relative mRNA expression levels in the anthers induced under high or low temperatures were investigated. The mRNA levels of the five proteins (accessions: Q0J889, Q69IN8, Q8LHG8, Q0INR8 and A0A0P0VZ12) exhibited better correlation with their corresponding protein levels: when the mRNA levels were up- or down-regulated, the abundance levels of their corresponding proteins were also increased or decreased compared to the control (Fig. 6). The mRNA and protein levels of Q53RJ5 and Q10CU4 were not correlated; when mRNA levels were up-regulated, protein abundance levels were decreased, suggesting that some other form of metabolic regulation occurred at the translation level. This suggests that changes in protein levels do not always parallel changes in the corresponding mRNA [16, 17].

## Discussion

### Fertility of AnnongS-1 was converted under heat stress

To explore the molecular mechanism of fertility conversion in AnnongS-1, we analyzed the DAPs in the anthers of AnnongS-1 plants grown under permissive and restrictive temperatures. After the AnnongS-1 plants were grown at 21 °C or > 26 °C, we analyzed the morphology of the anthers and the pollen grains. The anthers of AnnongS-1 plants grown at 21 °C developed normally. However, the anthers grown under high temperature conditions had a light yellow color and the pollen grains exhibited a wizened phenotype that could not be stained with I_2_-KI, which are features of abortive pollen grains (Fig. 1). These results demonstrated that AnnongS-1 became male sterile under the temperatures of > 26 °C, which is consistent with the observations of others [7]. Based on these data, the anthers collected from the rice plants grown under both temperature conditions were suitable for further comparative proteomic analysis to explore the fertility alteration mechanism.

In our present study, 89 DAPs were identified, including proteins differentially increased and decreased in abundance. These proteins have different biological functions and are involved with multiple cellular processes, suggesting that high temperature exerted extensive effects on AnnongS-1 anther cells. Notably, no Ub_L40_ proteins were identified in this study, which may be at least partially due to their low relative abundances, which reduced opportunities for detection. Although a high level of *Ub*_*L40*_ mRNA was closely associated with male sterility [7], until now there have been no reports showing that a high level of *Ub*_*L40*_ mRNA definitely results in a high level of Ub_L40_ protein. In fact, our experiments found that the levels of *Ub*_*L40*_ mRNA and Ub_L40_ protein are not necessarily parallel (unpublished observations). Therefore, we guess that *Ub*_*L40*_ genes affect AnnongS-1 fertility more at the mRNA level than at the protein level. On the other hand, in view of the fact that *Ub*_*L40*_*1* and *Ub*_*L40*_*4* overexpression only led to partial pollen abortion and *Ub*_*L40*_*1* and *Ub*_*L40*_*4* knockdown only partially restored male fertility [7], we speculate that sterility conversion in AnnongS-1 must involve other proteins besides Ub_L40_.

### High temperature stress adversely affected protein metabolism

After the DAPs were identified, we first noticed that the greatest number of proteins were enzymes involved in protein metabolism (Additional file 1: Table S1). Among the proteins with decreased abundance, eukaryotic translation initiation factor 1A is required for start site selection and a maximal rate of protein synthesis [18, 19]; Os08g0116500 protein shows 100% identity with PREDICTED: 60S acidic ribosomal protein P1 [*Oryza sativa* Japonica Group]. 60S acidic ribosomal protein P1 is not only involved with translation elongation [20], but also directly affects the phosphorylation of eIF2α by GCN2 kinase, which would in turn modulate translation in response to specific forms of stress [21]. The decrease in the abundance of these key regulatory proteins would reduce the start and elongation efficiencies of protein synthesis. Therefore, although the abundances of some other ribosomal proteins were increased, the overall efficiency of protein synthesis in the anthers could be decreased under high temperature conditions, which might be an important factor causing the sterility of AnnongS-1.

At the same time, although there are some protein folding-related DAPs such as calnexin and Os08g0197700 protein (heat shock 70 kDa protein BIP5) that were increased in abundance due to endoplasmic reticulum stress, the protein folding in the anther cells was still negatively affected by high temperature because many more important folding-related DAPs were decreased in abundance, such as DnaK protein (one of the top ten DAPs with decreased abundance, Table 1), peptidyl-prolyl cis-trans isomerase, a key enzyme for protein folding, 70 kDa heat shock protein and Os03g0804800 protein. Furthermore, proteasome-mediated protein degradation was enhanced, which might be relevant to the accumulation of the unfolded proteins. Of the three DAPs involved with proteasome-mediated protein degradation, proteins proteasome subunit alpha type-4-1 and proteasome subunit alpha type-1 were increased in abundance, while the abundance of Os02g0600100 protein, acting as enzyme regulator, was decreased, which suggested that the high temperature led to an increase in the proteasome-mediated protein catabolism and a decrease in the related regulatory ability of the anther cells. Besides, the decrease in the abundances of serine carboxypeptidase-like and carboxypeptidase would negatively influence protein metabolism and other related biological processes, including protein turnover, protein C-terminal processing, wound response and xenobiotic metabolism [22].

While protein synthesis was attenuated, the synthesis of some amino acids was also weakened due to a decrease in the abundances of relevant catalytic enzymes, such as serine hydroxymethyltransferase for glycine biosynthesis and D-3-phosphoglycerate dehydrogenase for L-serine biosynthesis. Besides, S-adenosylmethionine is an important methyl group donor for biosynthesis, including amino acid synthesis; nevertheless, S-adenosylmethionine synthase 1 was also decreased in abundance under high temperature conditions, which also might be one of the reasons why amino acid synthesis was weakened.

However, while translation and protein folding in the anther cells were adversely affected by high temperature, gene replication and transcription processes seemed to have been stimulated, because the abundant levels of Os09g0110400 protein (DNA-directed RNA polymerase II) (one of the top ten DAPs with increased abundance, Table 1) as well as histone-like protein (one of the top ten DAPs with increased abundance, Table 1) and histone H2A were elevated. Although histone H4 was decreased in abundance, the negative influence was probably limited because the decreased amplitude was smallest (HT/LT ratio 0.65, Additional file 2: Table S2) among all the proteins with decreased abundance under the high temperature conditions. Of note, the observation of transcription process enhancement under high temperature stress is consistent with the reports of Zhou et al. [7], who demonstrated that restrictive temperature significantly increased the *Ub*_*L40*_ mRNA levels. Under high temperature stress, several cytoskeletal proteins including actin-1, tubulin beta-4 chain, tubulin beta-3 chain and tubulin beta-1 chain were decreased in abundance, suggesting that the cytoskeletal organization was disturbed, which was supported by changes in the shape of the stamens grown under high temperature conditions (Fig. 1). In addition, the cytoskeleton also plays a fundamental role in assuring the cytoplasmic movement of organelles, vesicles and, in plants, the gametes. [23, 24]. Therefore, under conditions of high temperature, the disturbed cytoskeletal organization would adversely affect the cytoplasmic movement of a series of organelles and substances. On the other hand, the decrease in abundances of membrane steroid-binding protein 1 (acting as a coreceptor with SERL2 to enhance its endocytosis), putative nascent polypeptide associated complex alpha chain (targeting of ribosome-nascent polypeptide complexes) and Os07g0194000 protein (mediating vesicle-mediated transport and exocytosis) would further deteriorate the relevant cytoplasmic trafficking processes.

### High temperature negatively influenced carbohydrate and energy metabolism but seemed to favor lipid transfer

Analysis of the effect of high temperature on carbohydrate metabolism indicated that several key proteins had decreased abundance, including ribulose bisphosphate carboxylase small chain A for photosynthesis, succinate dehydrogenase [ubiquinone] flavoprotein subunit and aconitate hydratase for tricarboxylic acid cycle, and UDP-glucose 4-epimerase 3 for galactose metabolism, suggesting that photosynthesis, tricarboxylic acid cycle and galactose metabolism in the anthers were weakened under high temperature conditions. This finding was further supported by the increase in the abundance of OSJNBa0044M19.9 protein, a protein involved with nicotinic acid degradation. Nicotinic acid is the precursor for the synthesis of coenzymes NAD and NADP, which are essential for biosynthesis, catabolism, and energy metabolism. The adverse effects of high temperature on carbohydrate metabolism led to a decrease in the supply of NADH and FADH_2_, the substrates for oxidative phosphorylation.

Another mentionable enzyme involved with carbohydrate metabolism is Os09g0509200 protein (pyruvate dehydrogenase E1 component subunit beta). This protein is a component of the pyruvate dehydrogenase complex that catalyzes the overall conversion of pyruvate to acetyl-CoA and CO_2_. The increase in the abundance of Os09g0509200 protein under high temperature stress suggested that the production of acetyl-CoA was enhanced. Considering that the rate of the tricarboxylic acid cycle was lowered, as mentioned above, we postulated that the majority of the acetyl-CoA produced was not used for oxidation and energy supply, but rather for the biosynthesis of lipids, for example. In this study, four DAPs involved with lipid metabolism were increased in abundance. They are plant non-specific lipid-transfer proteins transferring phospholipids as well as galactolipids across membranes, which play roles in wax or cutin deposition in the cell walls of expanding epidermal cells and certain secretory tissues [25, 26]. These data suggest that, under high temperature conditions, the cellular processes involving lipid transfer and cell wall formation were active.

### High temperature altered metabolic regulation

High temperature not only affected the biosynthesis, catabolism, and energy metabolisms in the anthers of AnnongS-1, but also changed the metabolic regulatory system, as reflected by nine proteins participating in metabolic regulation being differentially increased or decreased in abundance. Of them, cold shock domain protein 2 and nascent polypeptide-associated complex subunit beta, participating in the regulation of transcription, were increased in abundance, while eukaryotic translation initiation factor 1A and Os08g0116500 protein, which is involved in translational elongation, were decreased in abundance. This finding is in agreement with the conclusion that transcription was enhanced whereas translation was weakened under high temperature conditions.

### Growth and development were impeded under high temperature conditions

Due to the adverse effects of high temperature on the substance and energy metabolism in the anthers of AnnongS-1, metabolism-based anther growth and development were disturbed. Xiao et al. [17] drew similar conclusions when they employed the proteomic strategy to study the young panicle proteome in the TGMS rice Zhu-1S. In our present work, we found that three DAPs closely related to growth and development showed decreased abundance, including Os09g0481000 protein, Os08g0413000 protein, and translationally-controlled tumor protein homolog, indicating that the high temperature impeded the normal growth and development of the pollen by decreasing the abundances of relevant proteins or enzymes. For example, BLAST analysis of Os09g0481000 protein indicated that Os09g0481000 protein has 100% identity with PREDICTED: BURP domain-containing protein 15 [*Oryza sativa* Japonica Group. BURP domain-containing protein 15 is closely related to pollen development. It is specifically expressed in the tapetum, connective and endothecium tissues of anthers [27, 28]. Its expression was observed during the late stage of anther development, starting at the tetrad stage. It reached a maximum level at the late vacuolated-pollen stage [27]. In addition, as a BURP domain-containing protein, Os09g0481000 may be important in the response to stress conditions [28].

### High temperature weakened the defense and stress ability of AnnongS-1

Our experimental results indicate that high temperature affected the defense and stress system of AnnongS-1 plants, with four relevant DAPs being increased in abundance and seven decreased in abundance. The four DAPs with increased abundance were Os12g0405700 protein, glutaredoxin-C6, Os08g0197700 protein, and elicitor-responsive protein 3. They were mainly involved in responses to cytokinin, cell redox homeostasis, or endoplasmic reticulum stress. The seven DAPs with decreased abundance were 70-kDa heat shock protein, Os01g0624000 protein, Os01g0916600 protein, superoxide dismutase [Cu-Zn] 1, peroxidase, Os01g0933900 protein, and DnaK protein. They participated in the responses to heat, oxidation, cold and other stressors, playing even more important roles in defense and various abiotic stresses. The decrease in abundances of these proteins seriously weakened the ability of AnnongS-1 to resist high temperature stress, because the tolerance of a rice cultivar to high temperature is due to highly increased abundances of heat shock protein, DnaKprotein, chaperon, peroxidase, and superoxide dismutase, among others [29–32]. For example, the 70-kDa heat shock protein is one of five types of heat shock proteins in plants [33]. As an essential regulator of protein (maintaining internal cell stability like proper folding protein and breakdown of unfolded proteins) [34], the 70-kDa heat shock protein is generally strongly increased in abundance by heat stress and some other kinds of stresses [35]. However, in our present study, this protein was decreased in abundance under high temperature conditions. The decrease in abundance of 70-kDa heat shock protein and other anther proteins involved with defense and stress might also be important factors that caused the male sterility of AnnongS-1. Overall, the defense and stress ability of AnnongS-1 was weakened under high temperature conditions, although there were several defense and stress-related proteins that had been increased in abundance.

## Conclusions

This study was the first comparative quantitative proteomic analysis of the anthers of TGMS line AnnongS-1 grown under permissive (low) (21 °C) and restrictive (high) temperatures (> 26 °C). The pollen grains formed under high temperature conditions were abortive while those formed under low temperature developed normally. Compared to the low temperature, high temperatures resulted in 89 proteins differentially accumulated in the anthers; 46 had increased abundance and 43 had decreased abundance. These DAPs are distributed in most of the subcellular compartments of anther cells and most have catalytic and/or binding molecular functions. GO analysis for biological process indicated that high temperature induction caused the fertility-sterility conversion mainly via adversely affecting the metabolism of protein, carbohydrate and energy, and decreasing the abundances of important proteins closely related to defense and stress, thus impeding the growth and development of the pollen and weakening the overall defense and stress ability of AnnongS-1. This work not only has deepened our understanding of the molecular mechanism underlying the fertility conversion in TGMS lines, but also provided new clues for further research.

## Methods

### Preparation of plant materials

The thermo-sensitive genetic male sterile AnnongS-1 seeds were obtained from Anjiang Agricultural School of Hunan Province, China. The plants were grown in plastic pots filled with soil and placed in the paddy field of the Hybrid Rice Research Base in Hunan Normal University, Hunan, China. When the flag leaves of the plants had emerged about 10 cm (about one week before anthesis), some of the pots with their plants were transferred into a shallow pool containing 21 °C water that was circulated through an industrial chiller. The others were left in the paddy field under continuous high temperature (> 26 °C). After 6 days, the young panicles of the plants were collected and stored under − 80 °C until extraction of proteins and nucleic acids. The fertility status of the anthers was determined by observing the shape and color of stamens, the number and shape of pollen grains, and the starch content with the help of light microscope and iodine staining.

### Protein extraction

The collected anthers were washed with a precooled PBS (phosphate buffered saline) buffer and placed in a precooled porcelain mortar. Liquid nitrogen was slowly added, followed by grinding. A lysis buffer (8 M urea, 50 mM NH_4_HCO_3_, pH 7.8) containing a protease inhibitor cocktail (Sigma-Aldrich) was added at a sample powder-to-lysis buffer volume ratio of about 1:8. After blending, the mortar was placed at 4 °C for 1 h in a refrigerator, followed by treatment with sonication to promote the cell lysis. After standing in the refrigerator for another 0.5 h, the extracted solution was centrifuged at 12000 g for 15 min at 4 °C. The supernatant was collected, and a six-fold volume of precooled acetone was added to precipitate the proteins. After centrifugation at 8000 g for 10 min at 4 °C, the pellet was collected and dried under vacuum and stored at − 80 °C until further use. Protein quantitation was carried out with the Bradford method, using BSA (bovine serum albumin) as the standard [36].

### Protein digestion and TMT labeling

The in-solution digestion and TMT labeling of the tryptic peptides were carried out according to the manufacturers’ protocols and related literature [29, 37, 38]. The proteins (100 μg each sample) were dissolved in 100 μL 45 mM TEAB (triethylammonium bicarbonate) and were reduced with 10 mM DTT at 55 °C for 1 h, and then were alkylated with 20 mM IAA at room temperature for 0.5 h in darkness. A six-fold volume of acetone pre-cooled at − 20 °C was added to the reaction mixture solution, followed by centrifugation at 8000 g for 10 min at 4 °C and removal of the supernatant. The pellet was suspended in 100 μL 100 mM TEAB and trypsin was added at an enzyme:protein ratio of 1:40, followed by overnight incubation at 37 °C. TMT labeling reagent (0.8 mg) dissolved in anhydrous ACN (acetonitrile) was added and the labeling reaction was allowed to proceed at room temperature for 1 h. Hydroxylamine (8 μL 5%) was added to stop the reaction. The experiments were performed in triplicate. In the first and the third repetitions, the anther proteins of the high temperature-treated sterile line plants were labeled with TMT 128, and those of low temperature-treated fertile line plants were labeled with TMT 129. In the second repetition, the labeling method was reversed, with former being labeled with TMT 129 and the latter with TMT 128. The comparative samples were equally mixed for quantitative analysis by LC-MS/MS (liquid chromatography-tandem mass spectrometry).

### Liquid chromatography-tandem mass spectrometry and bioinformatics

The mixed samples were analyzed using an Orbitrap Velos Pro mass spectrometer (Thermo Fisher Scientific, Bremen, Germany) coupled with an Easy-nLC system (Thermo Fisher Scientific, Odense, Denmark) according to the method of Na et al. [37]. Briefly, the peptide mixture was dissolved in 0.1% formic acid and separated on an EASY-Spray column (75 μm in diameter and 50 cm in length). The peptides eluted from the column were directly delivered into the mass spectrometer for MS and MS/MS analyses. The acquired spectra were analyzed and searched against the Uniprot *Oryza sativa* database by the SEQUEST algorithm in Proteome Discoverer 1.4 (Thermo Fisher Scientific). The main searching parameters were set as follows: trypsinolysis with up to two missed cleavage sites; mass error at 20 ppm for precursor ions and 0.8 Da for fragment ions, fixed modification of carbamidomethylation-cysteine, TMT 4-plex of lysine and N-terminus and variable modifications for methionine oxidation. After database searching, the peptide identifications were validated using the PeptideProphet algorithm; only those with high confidence (> 95%) were accepted. The FDR (false discovery rate) was set to 0.01. TMT signals showing at least 1.5-fold change in abundance were used for quantitative analysis of the identified proteins. The GO (gene ontology) annotations on the cellular component, molecular function and biological process of the DAPs were retrieved from the UniProt databases (http://www.uniprot.org) and the linked QuickGO (https://www.ebi.ac.uk/QuickGO). For proteins with incomplete GO annotations, BLAST analysis was performed and the GO annotations of the proteins with high homology (> 90% identity) were referenced. PSORT (http://www.psort.org) was employed to predict the subcellular locations of the proteins.

### Quantitative RT-PCR

The RNAs of representative DAPs were analyzed with quantitative RT-PCR (reverse transcription polymerase chain reaction). The total RNA extraction from the anthers, the first-strand DNA synthesis and qPCR (quantitative polymerase chain reaction) were performed according to the instructions of the corresponding commercial kits (TransGen Biotech, Beijing, China). Briefly, certain amounts of anthers were grinded in liquid in a mortar. For RNA isolation, TRIzol-chloroform extraction was performed, followed by isopropanol precipitation. RNase-free water was used to dissolve the extracted RNA for concentration and 260/280 ratio determinations. The cDNA was synthesized and then used as the template for PCR amplification of the selected genes with the primers listed in Additional file 2: Table S2. PCR amplification was performed under the following conditions: preincubation for 30 s at 94 °C, followed by 45 cycles of denaturation for 5 s at 94 °C, annealing and extension for 30 s at 55 °C. All samples were run at least in triplicates.

### Statistical analysis

We used IBM SPSS version 16.0 (Chicago: SPSS Inc.). A two-tailed paired t-test was used for the statistical analysis of differences between the control (21 °C conditions) and the test (> 26 °C conditions) plants. Differences were considered significant at *p* < 0.05.

## Additional files

Additional file 1: **Table S1** Information on the differentially abundant proteins in the anther of AnnongS-1 in response to restrictive temperature. (XLSX 29 kb)

Additional file 2: **Table S2** Primer sequences used for qPCR. (DOCX 20 kb)

## Abbreviations

ACN: Acetonitrile
BSA: Bovine serum albumin
DAPs: Differentially abundant proteins
FDR: False discovery rate
GO: Gene ontology
LC-MS/MS: Liquid chromatography-tandem mass spectrometry
MALDI-TOF: Matrix-assisted laser desorption/ionization-time of flight
PBS: Phosphate buffered saline
qPCR: Quantitative polymerase chain reaction
RT-PCR: Reverse transcription polymerase chain reaction
SDS-PAGE: Polyacrylamide gel electrophoresis
TEAB: Triethylammonium bicarbonate
TMT: Tandem mass tag
UbL40: Ubiquitin fusion ribosomal protein L40
