# Supplementary material for: A quantitative proteomic analysis of the molecular mechanism underlying fertility conversion in thermo-sensitive genetic male sterility line AnnongS-1
Source: BMC Plant Biol. 2019 Feb 11;19:65. doi: 10.1186/s12870-019-1666-5 (PMC6371510; doi:10.1186/s12870-019-1666-5)
Supplement: Supplementary file 2 — Table S2 Primer sequences used for qPCR. (DOCX 20 kb) [file 12870_2019_1666_MOESM2_ESM.docx]

**Table S2. Primer sequences used for qPCR**.

**Gene name Forward primer Reverse primer**

QOJ889 TACAACTGGAGCGTGAAG TAGCCTCAGTACCTTCTCT

Q69IN8 GAGTCCATTGTCCATCATAC GGTGTTCTTGTTCTTATCCA Q8LHG8 ATCAGCGGCAAGAAGAAG GAAGAACTCGGTGTCCTC

Q0INR8 ATCTACGACGTGAACTCC TTCATTCATCACAGGCTTG AOAOPOVZ12 GTTCTTCCTGTTGCTTGTA   TCCTTGAGTCCATCTTGA Q53RJ5 GAGAAGGAGGACTACGAG CTTGTAGACCTGCTTGATG Q10CU4 ACTACTCCTACTCTGTCCA  GAATGATGCCGTTGTTGA
